# Labour market marginalisation subsequent to suicide attempt in young migrants and native Swedes

**DOI:** 10.1007/s00127-017-1344-6

**Published:** 2017-02-20

**Authors:** T. Niederkrotenthaler, M. Wang, M. Helgesson, H. Wilcox, M. Gould, E. Mittendorfer-Rutz

**Affiliations:** 1 Suicide Research Unit, Department of Social and Preventive Medicine, Center for Public Health, Medical University of Vienna, Kinderspitalgasse 15, 1090 Vienna, Austria; 2 Department of Clinical Neuroscience, Division of Insurance Medicine, Karolinska Institutet, 17 177 Stockholm, Sweden; 3 Johns Hopkins School of Medicine, Baltimore, MD 21205 USA; 4 NYS Psychiatric Institute, Columbia University, New York, NY 10032 USA

**Keywords:** Suicide attempt, Migration, Labour market, Unemployment, Sweden

## Abstract

**Purpose:**

This study aimed to compare young individuals who differed in terms of birth region and history of suicide attempt regarding socio-demographic and healthcare factors, and with regard to their risks of subsequent unemployment, sickness absence and disability pension.

**Methods:**

Prospective cohort study based on register linkage of 2,801,558 Swedish residents, aged 16–40 years in 2004, without disability pension and with known birth country, followed up 2005–2011. Suicide attempters treated in inpatient care during 2002–2004 (*N* = 9149) were compared to the general population of the same age without attempt 1987–2011 (*N* = 2,792,409). Hazard ratios (HR) and 95% confidence intervals (CIs) for long-term unemployment (>180 days), sickness absence (>90 days), and disability pension were calculated with Cox regression, adjusted for several risk markers.

**Results:**

Compared to Swedish natives with suicide attempt, migrants of non-Western origin with attempt received less specialised mental healthcare. Distinct differences between native Swedes and migrants were present for the three labour market outcomes, but differences between migrant subgroups were inconsistent. As compared to native Swedes without attempts, non-European migrants with suicide attempt had adjusted HRs and CIs for subsequent unemployment 2.8 (2.5–3.1), sickness absence 2.0 (1.7–2.3) and disability pension 2.2 (1.8–2.6). Respective estimates for natives with suicide attempt were 2.0 (1.9–2.1); 2.7 (2.6–2.9) and 3.4 (3.2–3.6), respectively.

**Conclusions:**

Migrant suicide attempters receive less specialised mental health care before their attempt than native Swedes, and their marginalzation patterns are different. Healthcare and policy makers need to take the differential risk profile for migrant and native populations into account.

## Introduction

Suicide attempts represent a considerable public health problem in a number of countries worldwide [1]. Suicide attempts often have an early onset, are prone to repetition and are a risk factor for suboptimal social and functional outcomes, like poor academic achievements [2, 3]. In spite of these findings, there is a scarcity of research on the long term labour market outcomes associated with youth suicide attempt [4, 5]. Here, the literature on sickness absence and disability pension as measures of labour market marginalisation is particularly sparse [6].

Increasing global migration has led to dramatic demographic changes in Sweden, like in many other European countries [7]. To date, Sweden gives home to a multicultural society and around 15% of the population living in Sweden are first generation migrants [7]. While some 50 years ago, predominantly individuals with a European background formed the bulk of the workforce immigrants to Sweden, the last 30 years have seen increasing migration from refugees and non-European migrants with more diverse cultural and ethnic origins than the Swedish native population [7]. This diversity of migrant populations is reflected in differences with regard to access and use of healthcare [8], educational level [9], and prevalence of suicide attempt, [1] which all may impact on the risk of labour market marginalization [10].

Suicide attempt is first and foremost closely related to mental disorders [1]. In addition, we have shown in an earlier study that suicide attempt was an independent risk factor of marginalization beyond mental disorders [4]. The mechanisms linking suicide attempt in young adulthood to subsequent adverse health and social outcome are likely to be multifaceted. First, any association might be seen from a medical perspective, namely as a consequence of inherent deficits associated with suicide attempt and/or the underlying mental disorder. Young people with suicide attempt may not fully develop knowledge and competencies or psychological and cognitive capabilities [11]. Such competencies and capabilities are necessary for achieving certain levels of occupational attainment. An association between suicide attempt in young age and subsequent adverse outcome can also be viewed from a perspective on health care, treatment and rehabilitation needs of patients and to which extent these are met [12].

On the other hand, processes leading to poor social attainment in people with suicide attempt may not only be driven by factors inherently related to the underlying disease, but may also be affected by processes like social selection and self-selection [13]. More specifically, young people with suicide attempts may be less likely to receive social support or even be subject to discrimination and social exclusion and they may themselves develop low expectations related to occupational attainment [14]. Thus, the association between suicide attempt and subsequent adverse social outcomes is complex, and social selection processes and social causation processes might be strongly intertwined [15].

Work seems to be central to individual identity, social roles and social status [16, 17]. Particularly for people with mental disorders, which comprise the main risk factors for suicide attempt, work seems to provide opportunities to experience a sense of accomplishment, a structure for daily routine and the possibility of belonging to a social group through interactions with co-workers [18]. It is, therefore, of crucial importance to analyse the effect of suicide attempt on subsequent labour market marginalisation.

Previous findings also suggest that pathways to labour market marginalisation subsequent to youth suicide attempt differ for native Swedes and particularly non-European first generation immigrants [19]. It is, therefore, important to investigate different immigrant subgroups. These differences might, e.g. arise from disparities in socioeconomic status and access to health care between natives and different migrant subgroups [20]. Culture may determine the way migrants experience and express mental ill-health, reflect upon their needs for psychiatric care, cope with traumatic events and are affected by the stigma attached to mental ill-health [21]. Despite common aetiological features of suicidal behaviour across cultures, risk factors may also differ [22, 23].

Despite the described development and resulting challenges for labour market integration, research regarding mental health including suicidal behaviour, migration and labour market marginalisation among young individuals has been considered to be underdeveloped [24]. The aim of this study was to investigate differences in socio-demographic and healthcare factors between migrant subgroups with different regions of birth and the native population in young individuals with and without suicide attempt. We further aimed to analyse the association of suicide attempts in these subgroups compared to the native Swedish population with regard to unemployment, sickness absence and disability pension.

## Methods

The study cohort comprised all individuals alive, resident in Sweden, aged between 16 and 40 years on December 31st, 2004, without disability pension and with available information on migration status in 2004 (*n* = 2,801,558). This cohort was followed up for 7 years (2005–2011).

Register data was available for each individual retrospectively and prospectively up to 31st December 2011 from: (1) Statistics Sweden: age, sex, country of birth, education, area of residence, and length of unemployment from 1990 and onwards. (2) The Social Insurance Agency: sickness absence and disability pension (date) from 1994 and onwards. (3) The National Board of Health and Welfare: date and cause of in- and specialised outpatient care starting from 1987 and from 2001, respectively; and date of death from 1961 onwards.

Exposure was defined as individuals who were treated in inpatient-care following a suicide attempt during the three years preceding study entry, i.e. 2002, 2003 and 2004 (*N* = 9149). The reference group comprised individuals from the general population with the same inclusion criteria but without any inpatient-care due to suicide attempt during the study period (1987–2011, *N* = 2792 409).

### Diagnoses of mental and somatic disorders and suicide attempt

All diagnoses were defined by the corresponding codes of the International Classification of Diseases (ICD) versions 8, 9 and 10. Suicide attempts were defined based on ICD 10 X60-84 and Y10-34. In the inpatient care register, external codes are reported separately from the main and side diagnoses. We included events of undetermined intent in the final analysis in line with a number of previous papers [4, 10], to limit under-reporting and also to adjust for regional differences in ascertainment methods. A sensitivity analysis revealed the comparability of the estimates for the two outcome measures (i.e. X60-84 and Y10-34). Of note, the ICD-codes cover cases of self-injury that were intentional but not necessarily showing an intent to die. The definition applied here is consistent with the WHO multicentre study on suicidal behaviour [25].

### Covariates

Socio-demographic characteristics comprising country of birth, age, sex, area of residence, and educational level were measured in 2004 for individuals without suicide attempt and in the year before suicide attempt for individuals with suicidal behaviour. Age was categorised into following categories: 16–21, 22–26, 27–31, 32–36 and 37–40 years of age. Educational status was grouped in <9, 10–12 and >12 years of education. Categorisation of region of residence was based on small towns, medium sizes cities, big cities. Missing values for covariates were coded as separate categories. Health care characteristics, i.e. mental or somatic in- and specialised outpatient care were introduced in the analyses as four dichotomised variables. These variables were measured during the year prior to the suicide attempt, and in 2004 for those without attempt.

### The Swedish social insurance system

The Swedish unemployment insurance system is made up of basic insurance and voluntary income-related insurance. The basic insurance is granted to every resident over 20 years who is enrolled at the employment office and is carrying out a job-seeking plan. Also students can be provided unemployment benefits provided they complete a specific number of academic courses. All residents irrespective of country of birth above the age of 16 with an income from work or unemployment benefits, who have a reduced capacity to work either because of disease or injury are eligible for sickness benefits received from the Social Insurance Agency. During the first 14 days, employees get sick pay from their employers, and there is one qualifying day without benefits. A physician certificate is required after 7 days of self-certification. All people above 30 years of age who due to disease or injury have a permanently impaired work capacity can be granted temporary or permanent disability pension. Individuals 19–29 years of age can also receive disability pension due to reduced work capacity or failure to complete compulsory school. The data used here are based on benefits paid by the Social Insurance Agency [26].

### Migration status

Migration status was measured as being a first generation migrant and birth countries were grouped in regions as follows: Sweden (natives); EU-25 and other Western countries (includes rest of Northern Europe, USA, Canada, Australia and New Zealand); Europe outside EU-25 (including former Soviet Union and Turkey); and other world regions (Asia, Africa, Middle and South America). This approach is related to earlier classifications [10].

### Outcome measures

Long-term unemployment and long term sickness absence were defined as having more than 180 and more than 90 registered days in a given year during follow-up, respectively [4, 27]. Disability pension was defined based on presence of a disability pension grant during follow-up.

### Statistical methods

We performed Chi-square tests to investigate any differences in socio-demographic and health care characteristics between the native population and migrant subgroups with different regions of birth in individuals with and without suicide attempt. Cox regression was used to calculate hazard ratios (HRs) and 95% confidence intervals (CIs) for the risk of subsequent labour market marginalisation (i.e. long term unemployment, long term sickness absence and disability pension). HRs were calculated for population groups that differed with regard to their region of birth and the presence of a suicide attempt, with native Swedes without suicide attempt as the reference group. Analyses were stepwise adjusted, first for sex, age, area of residence and education and then for the health care covariates. Censoring was due to emigration to a foreign country, death, or end of follow-up, whichever came first. In the analyses of unemployment and sickness absence as outcomes, censoring was also due to disability pension. Interaction of suicide attempt with region of birth, was tested using the partial likelihood ratio test.

### Ethical statement

Ethical approval for this study was obtained from the Regional Ethical Review Board, Karolinska Insitutet, Stockholm. The ethical review board approved the study and waived the requirement that informed consent of research subjects should be obtained.

## Results

In total, 9149 individuals had inpatient care due to attempted suicide between 2002 and 2004 when aged 14–40 years. Tables 1 and 2 shows descriptive statistics of the study population per region of birth country without and with suicide attempt, respectively. In both populations, all covariates differed significantly between natives and the migrant subgroups for all covariates (*p* < 0.001). Migrants without suicide attempt were generally more often women and older than native Swedes (Table 1). Table 1 further indicates that migrants from outside of the EU25/Western countries and from other world regions had more specialised mental and somatic health care. Moreover, migrants had higher levels of subsequent labour market marginalisation in terms of long-term unemployment, long-term sickness absence and disability pension, particularly those from Europe outside the European Union and other world regions.

**Table 1:** Descriptive statistics of individuals without suicide attempt 1987–2011 registered in Sweden and aged 16–40 years of age in 2004, stratified by migration status

Characteristics	Total *n* (%)	Sweden *n* (%)	EU25 and other Western *n* (%)	Europe outside EU25 *n* (%)	Other world regions *n* (%)	Chi^2^ *p*
Socio-demographic factors^a^						
Sex						<0.001
Women	1,357,187 (48.6)	1,145,095 (48.2)	56,617 (49.0)	47,266 (51.1)	108,209 (52.2)	
Men	1,435,222 (51.4)	1,232,222 (51.8)	58,877 (51.0)	45,141 (48.9)	98,982 (47.8)	
Age group (years)						<0.001
16–21	6,355,178 (22.8)	565,229 (23.8)	11,006 (9.5)	19,198 (20.8)	40,084 (19.3)	
22–26	507,777 (18.2)	431,469 (18.1)	18,052 (15.6)	17,201 (18.6)	41,055 (19.8)	
27–31	555,522 (19.9)	465,528 (19.6)	26,596 (23.0)	18,072 (19.6)	45,326 (21.9)	
32–36	587,870 (21.1)	490,920 (20.7)	30,991 (26.8)	20,263 (21.9)	45,696 (22.1)	
37–40	505,723 (18.1)	424,171 (17.8)	28,849 (25.0)	17,673 (19.1)	35,030 (16.9)	
Health care factors^a^						
Previous mental outpatient care						<0.001
Yes	34,411 (1.2)	28,799 (1.2)	1251 (1.1)	1,533 (1.7)	2828 (1.4)	
No	2,757,998 (98.8)	2,348,518 (98.8)	114,243 (98.9)	90,874 (98.3)	204,363 (98.6)	
Previous mental inpatient care						<0.001
Yes	8,940 (0.3)	7,272 (0.3)	383 (0.3)	333 (0.4)	952 (0.5)	
No	2,783,469 (99.7)	2,370,045 (99.7)	115,111 (99.7)	92,074 (99.6)	206,239 (99.5)	
Previous somatic outpatient care						<0.001
Yes	736,883 (26.4)	620,654 (26.1)	26,492 (22.9)	26,545 (28.7)	63,192 (30.5)	
No	2,055,526 (73.6)	1,756,663 (73.9)	89,002 (77.1)	65,862 (71.3)	143,999 (69.5)	
Previous somatic inpatient care						<0.001
Yes	187,924 (6.7)	155,755 (6.6)	7143 (6.2)	6951 (7.5)	18,075 (8.7)	
No	2,604,485 (93.3)	2,221,562 (93.4)	108,351 (93.8)	85,456 (92.5)	189,116 (91.3)	
Outcome measures^b^						
Unemployment	317,462 (29.8)	226,792 (26.4)	13,931 (36.7)	22,615 (44.4)	54,124 (45.8)	
Sickness absence	151,892 (18.3)	126,532 (17.7)	5969 (20.2)	7146 (23.3)	12,245 (21.6)	
Disability pension	56,307 (2.0)	45,834 (1.9)	2132 (1.8)	3073 (3.3)	5268 (2.5)	

^a^These variables were measured during the year prior to the suicide attempt, and in 2004 for those without attempt

^b^Measured during follow-up: 2005 to 2011

**Table 2:** Descriptive statistics of individuals with suicide attempt 2002–2004 registered in Sweden and aged 16–40 years of age in 2004, stratified by migration status

Characteristics	Total *n* (%)	Sweden *n* (%)	EU25 and other Western *n* (%)	Europe outside EU25 *n* (%)	Other world regions *n* (%)	Chi^2^ *p*
Socio-demographic factors^a^						
Sex						<0.001
Women	5947 (65.0)	4789 (63.4)	256 (64.5)	217 (72.8)	685 (68.4)	
Men	3202 (35.0)	2664 (35.7)	141 (35.5)	81 (27.2)	316 (31.6)	
Age group (years)						<0.001
16–21	3356 (36.7)	2873 (38.5)	83 (20.9)	91 (30.5)	309 (30.9)	
22–26	2226 (24.3)	1862 (25.0)	53 (13.4)	64 (21.5)	247 (24.7)	
27–31	1442 (15.8)	1085 (14.6)	87 (21.9)	62 (20.8)	208 (20.8)	
32–36	1135 (12.4)	875 (11.7)	78 (19.6)	46 (15.4)	136 (13.6)	
37–40	990 (10.8)	758 (10.2)	96 (24.2)	35 (11.7)	101 (10.1)	
Health care factors^a^						
Previous mental outpatient care						<0.001
Yes	899 (9.8)	765 (10.3)	45 (11.3)	12 (4.0)	77 (7.7)	
No	8250 (90.2)	6688 (89.7)	352 (88.7)	286 (96.0)	924 (92.3)	
Previous mental inpatient care						<0.001
Yes	982 (10.7)	859 (11.5)	38 (9.6)	15 (5.0)	70 (7.0)	
No	8167 (89.3)	6594 (88.5)	359 (90.4)	283 (95.0)	931 (93.0)	
Previous somatic outpatient care						<0.001
Yes	4493 (49.1)	3682 (49.4)	197 (49.6)	122 (40.9)	492 (49.2)	
No	4656 (50.9)	3771 (50.6)	200 (50.4)	176 (59.1)	509 (50.8)	
Previous somatic inpatient care						<0.001
Yes	1434 (15.7)	1177 (15.8)	68 (17.1)	35 (11.7)	154 (15.4)	
No	7715 (84.3)	6276 (84.2)	329 (82.9)	263 (88.3)	847 (84.6)	
Outcome measures^b^						
Unemployment	2048 (34.2)	1525 (32.6)	104 (41.1)	93 (43.3)	289 (40.1)	
Sickness absence	1587 (39.0)	1345 (39.7)	65 (37.8)	55 (34.6)	133 (35.2)	
Disability pension	1571 (17.2)	1330 (17.8)	81 (20.4)	29 (9.7)	131 (13.1)	

^a^These variables were measured during the year prior to the suicide attempt, and in 2004 for those without attempt

^b^Measured during follow-up: 2005–2011

With regard to individuals with suicide attempt (Table 2), the distributions across sex and age resembled the distributions in non-attempters. There were no considerable differences in the proportions of specialised somatic health care before suicide attempt between migrants and native Swedes with suicide attempt. With regard to previous specialised mental health care, however, suicide attempters from Europe outside EU25 and from other world regions were less likely to have such healthcare as compared to native Swedish suicide attempters. The proportions of suicide attempters with subsequent long-term unemployment were higher among migrants, whereas the proportions of attempters receiving sickness absence or disability pension were lower compared to native Swedes, with exception of higher rates of disability pension in migrants from EU25 and other Western countries.

There were statistical interactions between birth regions and the exposure variable (suicide attempt) with regard to all outcome variables (*p* < 0.001). Table 3 shows HRs and CIs for the three different outcome measures for categories with or without suicide attempt and for different migration statuses.

**Table 3:** Hazard ratios (HR) and 95% confidence intervals (CI) for unemployment, sickness absence, and disability pension 2005–2011 in relation to suicide attempt in 2002–2004 and regions of country of birth

Long-term unemployment		Crude	Model 1	Model 2
No suicide attempt^a^	Sweden	1	1	1
	EU25/Western	1.42 (1.40,1.45)	1.36 (1.33, 1.38)	1.36 (1.34, 1.39)
	Europe outside EU25	2.82 (2.78, 2.86)	2.47(2.43, 2.51)	2.46 (2.42, 2.49)
	Other world regions	3.08 (3.06, 3.11)	2.74 (2.70, 2.77)	2.71 (2.68, 2.75)
Suicide attempt	Sweden	2.56 (2.44, 2.69)	2.29 (2.18, 2.41)	1.98 (1.88, 2.08)
	EU25/Western	3.49 (2.88, 4.23)	3.04 (2.51, 3.68)	2.67 (2.21, 3.24)
	Europe outside EU25	3.89 (3.18, 4.77)	3.47 (2.83, 4.25)	3.24 (2.64, 3.98)
	Other world regions	3.61 (3.22, 4.05)	3.11 (2.77, 3.49)	2.80 (2.50, 3.15)

Long-term sickness absence		Crude	Model 1	Model 2
No suicide attempt^a^	Sweden	1	1	1
	EU25/Western	1.05 (1.03, 1.08)	0.98 (0.96, 1.01)	1.02 (1.00, 1.05)
	Europe outside EU25	1.49 (1.46, 1.53)	1.34 (1.30, 1.37)	1.34 (1.30, 1.37)
	Other world regions	1.15 (1.13, 1.17)	1.04 (1.02, 1.07)	1.04 (1.02, 1.06)
Suicide attempt	Sweden	3.96 (3.75, 4.18)	3.88 (3.67, 4.09)	2.69 (2.55, 2.85)
	EU25/Western	3.84 (3.01, 4.89)	2.99 (2.34, 3.81)	2.21 (1.73, 2.82)
	Europe outside EU25	3.06 (2.28, 4.12)	2.71 (2.01, 3.64)	2.36 (1.75, 3.17)
	Other world regions	2.79 (2.36, 3.31)	2.53 (2.14, 3.01)	1.96 (1.66, 2.33)

Disability pension		Crude	Model 1	Model 2
No suicide attempt^a^	Sweden	1	1	1
	EU25/Western	1.05 (1.00, 1.09)	0.63 (0.60, 0.66)	0.71 (0.68, 0.74)
	Europe outside EU25	1.76 (1.69, 1.82)	1.13 (1.08, 1.17)	1.09 (1.05, 1.13)
	Other world regions	1.36 (1.32, 1.40)	0.81 (0.79, 0.84)	0.81 (0.79, 0.84)
Suicide attempt	Sweden	10.26 (9.72, 10.84)	7.90 (7.46, 8.37)	3.38 (3.18, 3.59)
	EU25/Western	12.49 (10.04,15.53)	7.18 (5.77, 8.93)	2.82 (2.26, 3.51)
	Europe outside EU25	5.38 (3.74, 7.74)	2.64 (1.83, 3.80)	1.92 (1.33, 2.77)
	Other world regions	7.35 (6.19, 8.73)	4.03 (3.39, 4.79)	2.20 (1.85, 2.61)

Model 1 adjusted for age, sex, area of residence, education

Model 2 like Model 1 and additionally adjusted for previous inpatient and specialised outpatient care due to somatic and mental disorders

^a^Reference group without suicide attempt 1987–2011

### Long-term unemployment

For individuals without suicide attempt, HRs for long-term unemployment were in a gradient pattern higher for migrants from the EU25 and other Western countries (HR 1.4), from Europe outside the EU25 (HR 2.5) and from other world regions (HR 2.7) compared to natives in the multivariate analyses adjusting for age, sex, area of residence, educational level and previous inpatient and specialised outpatient care due to somatic and mental disorders (Table 3). Primarily socio-demographic factors made some contribution in decreasing estimates in the adjusted models, but the associations remained significant. For those with suicide attempt, the described patterns were comparable but estimates were higher in the multivariate analyses: natives (HR 2.0), EU25 and other Western countries (HR 2.7), Europe outside EU25 (HR 3.2) and other world regions (HR 2.8), when compared to natives without suicide attempt (Table 3). Estimates for individuals with suicide attempt were attenuated both after adjustment for socio-demographics and health care variables in the multivariate analyses.

### Long-term sickness absence

In the multivariate analyses, the risks for long-term sickness absence were similar for migrants without suicide attempt from the EU25 and other Western countries (HR 1.0) and from other world regions (HR 1.0), but slightly higher for European migrants from outside the EU25 (HR 1.3) compared to natives without suicide attempt. Estimates were somewhat attenuated after adjustment for socio-demographic factors. For migrants with suicide attempt, HRs for subsequent sickness absence were somewhat lower than those of native Swedes with suicide attempt: natives (HR 2.7), EU25 and other Western (HR 2.2), Europe outside the EU (HR 2.4) and for migrants from other world regions (HR 2.0). Adjustment for socio-demographics and particularly health care variables attenuated HRs for individuals with suicide attempt in the multivariate analyses.

### Disability pension

For individuals without suicide attempt, HRs for disability pension were lower for migrants from the EU25 and other Western countries (HR 0.7) and from other world regions (HR 0.8), and similar for migrants from Europe outside the EU25 (HR 1.1) compared to natives, in the multivariate analyses. Primarily socio-demographic factors made some contribution in decreasing estimates in the adjusted models. For those with suicide attempt, HRs for migrants were lower than HRs for native Swedes with suicide attempt, but considerably higher as compared to individuals without suicide attempt: natives (HR 3.4), EU25 and other Western countries (HR 2.8), Europe outside the EU25 (HR 1.9), and migrants born in other world regions (HR 2.2). Estimates for individuals with suicide attempt were attenuated after adjustment for both socio-demographics and health care variables in the multivariate analyses, with strongest effects of previous specialised health care, which was particularly relevant to the native, EU25 and other Western populations (Table 3).

## Discussion

### Main findings

Compared to Swedish natives, migrant groups without suicide attempt received more specialised mental healthcare, but migrants from outside the Western countries with attempt received less such care than Swedish natives with suicide attempt. The risk of labour market marginalization varied with regard to previous suicide attempt, region of birth country, and specific labour market marginalisation outcome. For individuals without suicide attempt, all migrant groups had higher risks for long-term unemployment and most had similar risks for long-term sickness absence and lower risks for disability pension as compared to natives. A suicide attempt was associated with higher estimates for all three outcome measures in all groups. For individuals with suicide attempt, all migrant groups had higher risk estimates than natives for subsequent unemployment, and lower estimates for sickness absence and disability pension, as compared to natives. No consistent pattern between different migrant groups with regard to the three outcome measures was observed.

Compared to natives without suicide attempt, the risk for long-term unemployment was higher for migrant subgroups without suicide attempt. The more distant the culture of the country of birth of the respective migrant group was, the higher were the risk estimates. This is in line with previous research suggesting that non-European migrants have a higher risk of unemployment than other migrants and natives [28]. Underlying mechanisms might be both related to post-migration difficulties including psychosocial acculturation problems and ethnical discrimination, but also differences in educational level [20]. In turn, these socio-economic disadvantages of migrants in the host country compared to the native population might be associated with access to lower paid jobs characterised by worse psychosocial environment and higher job insecurity [20].

Presence of a suicide attempt generally resulted in even higher risk estimates for subsequent unemployment, with generally similar patterns across migrant subgroups as seen in groups without suicide attempt. These findings contribute to the current literature on suicide attempt being a risk factor for subsequent unemployment [4]. The results suggest that the health selection due to a suicide attempt, i.e. the consequences of the deficits in social and occupational functioning associated with the mental disorder underlying the suicide attempt and/or the physical injuries associated with the suicidal act, may act similarly regardless of migration status [29]. We found one exception to this general pattern: migrants from other world regions had similar risk estimates for subsequent long-term unemployment regardless of a suicide attempt. An explanation for this is that migrants from other world regions without a suicide attempt already had a high risk of unemployment, i.e. most prominent difficulties to establish themselves on the labour market. A suicide attempt did not seem to contribute to exacerbate this risk.

Risk patterns for subsequent sickness absence and disability pension differed from those with regard to long-term unemployment. For individuals without suicide attempt, there were similar or only slightly increased risk estimates for the different migrant groups with regard to subsequent sickness absence, and there were even lower risk estimates for most migrant subgroups with regard to subsequent disability pension. To the best of our knowledge, this has not been reported to date. In those individuals with previous suicide attempt, all migrant groups had higher risks for subsequent sickness absence and disability pension compared to natives without suicide attempt. However, in contrast to the patterns observed for unemployment, risk estimates were highest for natives with suicide attempt for both sickness absence and disability pension. Here, estimates were significantly lower for migrants from other world regions with regard to both outcome measures. This is in line with previous findings which show that young individuals with suicide attempt born outside Europe had a higher risk of unemployment but a lower risk of disability pension, as compared to the Swedish-born population [19]. However, findings are inconsistent with other results indicating that adults with a depressive disorder had higher risk estimates for subsequent disability pension if their country of birth was outside Europe [30]. Discrepancies might stem from differences in the study populations, i.e. differences between suicide attempters and patients with depressive disorders, or from differences in age groups. More research is warranted to elucidate these discrepancies in findings.

Earlier studies on suicide attempt have shown cultural differences in the prevalence and severity of suicide attempts [1, 31]. Despite common aetiological features of suicidal behaviour across cultures, risk factors may also differ [32]. For example, lower rates of mental ill-health and a higher likelihood to respond impulsively to stressful life events have been reported for suicidal behaviour of Asian women than what is known from the literature based on women in Western countries [32]. It is possible that if suicide attempts are less related to mental disorders in some migrant groups, some migrant groups might, therefore, be less likely to need a disability pension or a prolonged sickness absence. Another explanation might be connected to higher levels of stigma among some migrant groups with regard to accessing the disability pension and/or taking an extended sickness absence [34, 35].

In addition, some migrant groups may be less informed about the Swedish welfare system and the accessibility of sickness absence and disability pension. Moreover, formal requirements for receiving sickness absence include a minimum level of salary which might not be reached among some migrant groups. Disability pension, in turn, does not have this requirement, but is often preceded by sickness absence, which means that the pathway to disability pension among some migrant groups may deviate from those in natives.

Experiences of discrimination may play a role in the identified associations. Discrimination is likely to be more prevalent against migrants [21], and may also be associated with an increased risk of suicide attempt. Additionally, discrimination may be at play in terms of greater risk for unemployment, reduced access to the disability pension and a reduced capacity to take a prolonged absence from work.

Moreover, discrimination in the health care setting, as well as with regard to access of care, and adequate treatment, likely plays a role in the identified associations. Here, potential discrepancies can be discussed in the framework of differences in access to care, in clinical manifestation and symptomatology of the underlying disease and consequently in its diagnostics as well as differences in care after a suicide attempt [21]. Adequate care following suicidal behaviour in immigrants/refugees might be hampered by language barriers, as well as the lack of competence in transcultural psychiatry and psychology in the health care settings of the host country [22].

In this context, it is interesting that natives and EU25 and other Western countries-born individuals showed a strong reduction of risk estimates for disability pension when adjusting for their healthcare characteristics, which was less pronounced in respective analyses for migrant populations from European countries outside the EU and other world regions. This finding may again indicate that a suicide attempt is more strongly related to mental and somatic disease in natives and individuals from Western countries, and/or it may indicate that migrants from birth countries in Europe outside the EU and from other world regions are less frequently seeking healthcare before their suicide attempt. This is supported by our findings that specialised mental healthcare was lower in suicide attempters with birth countries outside EU25 and other world regions than in natives, while such care was higher in these migrants without suicide attempt.

### Strengths and limitations

Strengths of the present study are the prospective design based on national register data. These registers have practically no dropouts. Several registers have been evaluated and have shown high data quality, with, e.g. 1.2 and 0.8% missing main diagnoses reported for the National Patient Register and the Causes of Death Register, respectively [36]. Registers of the Social Insurance Agency with data on sickness absence and disability pension indicate received benefits and have generally been considered to be of good quality [26].

The present study has also limitations. First, due to data availability on national level, only individuals hospitalized for suicide attempt were included. Around a quarter of suicide attempters have been estimated to receive inpatient care, therefore, the present findings can only be generalised to more severe cases of suicide attempts [37].

Another limitation is that the occurrence of unemployment, sickness absence, and disability pension is affected by regional and temporal changes in social insurance policies and in fluctuations in economic development [38, 39]. Therefore, studies from different birth cohorts and from different countries are needed to assess potential differences in patterns. Finally, heterogeneous groups of migrants were collapsed into categories. This was partly to guarantee sufficient power for the respective analyses and partly due to data availability. This particularly applies to the group “other world regions”. Heterogeneous patterns within this collapsed group seem likely and future studies are warranted to elucidate differences in patterns of labour market marginalisation in this group.

## Conclusion

While migrant groups of non-Western origin did not generally receive less healthcare than natives, particularly suicide attempting migrants received less specialised mental healthcare as compared to their native counterparts. Research that investigates the reasons for identified inequalities in specialised healthcare received by migrant suicide attempters is warranted. With regard to subsequent labour market marginalisation, suicide attempt increased the risk of labour market marginalisation in both natives and Swedes regardless of outcome measures, and migrant groups were generally more prone to long-term unemployment and less prone to subsequent sickness absence and disability pension compared to their native counterparts. Healthcare and policy makers need to take the differential risk profile for migrant and native populations into account.
